# A scalable framework for single-cell eQTL mapping uncovers genetic regulators of meat production traits in pigs

**DOI:** 10.1186/s40104-026-01452-5

**Published:** 2026-06-28

**Authors:** Qiqi Zhang, Qi Bao, Lingsen Zeng, Zhicheng He, Zhen Wang, Cong Li, Guoqiang Yi

**Affiliations:** 1 College of Animal Science and Technology, Northwest A&F University, Xianyang, 712100 China; 2 State Key Laboratory of Genome and Multi-Omics Technologies, Shenzhen Branch, Guangdong Laboratory of Lingnan Modern Agriculture, Key Laboratory of Livestock and Poultry Multi-Omics of MARA, Agricultural Genomics Institute at Shenzhen, Chinese Academy of Agricultural Sciences, Shenzhen, 518124 China; 3 Animal Breeding and Genomics, Wageningen University & Research, Wageningen, 6708 PB The Netherlands; 4 Bama Yao Autonomous County Rural Revitalization Research Institute, Bama, 547500 China

**Keywords:** Cell-type-specific regulation, eQTLs, Pig, sc/snRNA-seq, Skeletal muscle

## Abstract

**Background:**

Genetic determinants that regulate molecular phenotypes and complex traits often act in a highly context-dependent manner, and the underlying cell-type-specific regulatory mechanisms remain incompletely understood.

**Results:**

In this study, we analyzed 42 single-cell and single-nucleus RNA sequencing (sc/snRNA-seq) datasets from pig skeletal muscle. Through systematic benchmarking, we optimized a robust workflow for SNP calling and genotype imputation tailored to sc/snRNA-seq data, achieving high accuracy and computational efficiency. We constructed a comprehensive single-cell atlas of skeletal muscle that delineates cellular components and developmental trajectories, and identified 5,020 significant single-cell expression quantitative trait loci (eQTLs). By integrating phenotypic data from the PigGTEx project and performing phenome-wide association studies (PheWAS), we further pinpointed three candidate loci significantly associated with meat quality and growth traits whose effects were dependent on cell type.

**Conclusions:**

This work offers a scalable computational framework for single-cell eQTL mapping and characterizes cell-type-specific associations between genetic variation and gene expression relevant to economically important traits in livestock, thereby providing functional context in particular for non-coding variants implicated by GWAS and helping to inform genomic selection and precision genome editing.

**Supplementary Information:**

The online version contains supplementary material available at 10.1186/s40104-026-01452-5.

## Introduction

Genome-wide association studies (GWAS) have shown that the majority of loci associated with common diseases and complex traits reside in non-coding regions [1]. These variants often alter gene expression by modulating the regulatory activity and chromatin interactions of non-coding elements such as enhancers and promoters, thereby contributing to phenotypic variation and disease risk [2, 3]. To functionally interpret such non-coding loci, quantitative trait locus analyses of molecular phenotypes, particularly expression quantitative trait loci (eQTLs), systematically link genetic variants to their regulatory and transcriptional consequences [4]. These approaches complement GWAS by helping to resolve how non-coding genetic variation shapes downstream molecular and organismal traits. Together, this underscores the importance of systematically characterizing the context-dependent regulatory effects of genetic variants. At the population level, the human Genotype-Tissue Expression (GTEx) project [5] and GTEx-analogous efforts in livestock (e.g., PigGTEx [6]) have generated extensive tissue-level eQTL maps [7], typically using large volumes of RNA-seq data from bulk tissues. However, tissues comprise multiple cell types, and the resulting heterogeneity in cellular composition can mask expression differences across cell types and states [8, 9].

By quantifying gene expression at single-cell resolution, sc/snRNA-seq profiling reveals the transcriptional states of individual cells [10–12] and enables direct eQTL mapping at the cellular level. Previous human studies have demonstrated that single-cell eQTL analyses can uncover cell-type-specific and state-dependent regulatory effects that are obscured in bulk eQTL analyses and can delineate genetic effects along dynamic processes such as differentiation and immune activation [7, 13–15]. However, because generating single-cell datasets with matched genotype information at the cohort level is prohibitively expensive, many studies have instead relied on deconvolution frameworks (e.g., CIBERSORTx, MuSiC) to infer cell-type proportions or cell-type-specific expression from bulk RNA-seq data before eQTL analysis [16, 17]. Such approaches are sensitive to reference atlases, inter-individual variability, and model specification, which can limit accuracy and interpretability, and therefore direct quantification and association testing at the single-cell level is preferable [18, 19].

Pigs are economically critical livestock valued for traits including growth rate, feed efficiency, and meat quality, and they also serve as vital biomedical models due to their physiological proximity to humans [20, 21]. At the tissue level, porcine multi-omics and regulatory resources across multiple organs have enabled cross-tissue eQTL maps that link genomic variation to traits of economic importance [6, 22]. More recently, high-throughput single-cell and single-nucleus atlases in pigs have expanded rapidly across various developmental stages and tissues, refining our understanding of muscle-resident lineages (e.g., satellite cells, fibro/adipogenic progenitors, and type I/II myonuclei), stromal and vascular compartments, and immune subsets [6, 23]. Because many economically relevant traits and health endpoints are driven by processes confined to particular cell types, including myogenesis, adipogenesis, angiogenesis, and inflammation, resolving regulatory variants at cellular resolution can provide in-depth molecular insights [24–26] and help nominate actionable targets for genomic selection and precise genome editing. Despite this emerging foundation, single-cell eQTL (sc-eQTL) resources in pigs remain scarce.

In this study, we investigated potential cell-type-specific regulatory mechanisms using 42 sc/snRNA-seq datasets from porcine skeletal muscle, combining newly generated and publicly available data. Following systematic benchmarking, we selected CellSNP-lite [27] as a high-precision genotyper and constructed a high-concordance imputation workflow. By integrating a single-cell atlas of skeletal muscles, we identified 5,020 significant sc-eQTLs. By further integrating PigBiobank PheWAS evidence [28], we highlighted three candidate loci with significant associations with meat quality and growth traits by cell-type-dependent effects. Collectively, this study presents a scalable framework for single-cell eQTL analysis and provides cell-type-resolved insights into how non-coding variation may influence meat production traits. This integration nominates these variants as high-priority candidates whose regulatory effects may contribute to phenotypic variation.

## Materials and methods

### Sample collection

We sampled six Meishan pigs and generated eight snRNA-seq libraries for three tissues, including longissimus dorsi muscle (*n* = 6), adipose (*n* = 1), and liver (*n* = 1). For SNP-caller benchmarking, five individuals among all pigs were subjected to paired whole-genome sequencing (WGS). The skeletal muscle dataset used for *cis*-eQTL mapping integrated (i) six newly generated snRNA-seq data from longissimus dorsi tissues and (ii) 36 public skeletal-muscle sc/snRNA-seq samples from eight independent studies (Table S1) [29–37]. Both pseudo-bulk and sc-eQTL analyses were performed on this combined dataset. For all samples, detailed metadata were systematically curated, including study origin, breed, age (developmental stage), tissue source, sequencing platform, and sequencing technology, and are summarized in Table S1. The snRNA-seq data from liver and adipose were used exclusively for benchmarking and validating the RNA-derived SNP calling and genotype imputation pipelines across different transcriptional contexts.

### Whole-genome resequencing library preparation and sequencing

WGS libraries were prepared by Berry Genomics (Beijing, China) following the standard Illumina protocol. Briefly, genomic DNA was fragmented to ~350 bp using ultrasonication. After end repair, A-tailing, and adapter ligation, libraries were size-selected, purified, and PCR-enriched. Library quality was assessed using an Agilent 2100 Bioanalyzer and quantitative PCR. Qualified libraries were sequenced on an Illumina NovaSeq 6000 platform to generate 150-bp paired-end reads. Raw data were processed with instrument software for base calling and demultiplexing to obtain FASTQ files for downstream analysis.

### Single-nucleus RNA-seq library preparation and sequencing

SnRNA-seq libraries were prepared by Berry Genomics (Beijing, China) following the 10 × Genomics Chromium protocol. Isolated nuclei were encapsulated with barcoded gel beads using the Chromium Controller. Following reverse transcription, emulsions were broken and barcoded cDNA was purified and PCR-amplified. The amplified cDNA was used to construct indexed 3′ gene-expression libraries. Libraries that passed quality control were sequenced on an Illumina NovaSeq 6000 platform to generate 150-bp paired-end reads. Read 1 (28 bp) contained the 16-bp cell barcode and the 12-bp unique molecular identifier (UMI).

### SNP calling from whole-genome sequencing data

WGS data from individuals matched to the snRNA-seq cohort underwent quality assessment with FastQC (v0.12.1) [38]. The reads were trimmed by fastp (v0.23.0) [39] with default parameters and clean paired-end reads were aligned to the reference genome using the BWA-MEM algorithm (v0.7.18) [40], and generated alignments were converted to coordinate-sorted, indexed BAMs with SAMtools (v1.13) [41].

PCR duplicates were identified and removed using the MarkDuplicates module of GATK (v4.5.0.0) [42]. Variant calling was then performed using GATK HaplotypeCaller. Variants were filtered using the recommended hard-filtering criteria: for SNPs, *QD* > 2.0, *FS* < 60.0, *MQ* > 40.0, *SOR* < 4.0, *MQRankSum* > −12.5, and *ReadPosRankSum* > −8.0; for insertions and deletions (Indels), *QD* > 2.0, *FS* < 200.0, and *SOR* < 10.0. Functional annotation of the resulting variants was performed using SnpEff [43].

### Single-cell/nucleus RNA-seq data preprocessing

Raw FASTQ files for each library were processed independently using CellRanger (v9.0.1) (https://github.com/10XGenomics/cellranger) and, where applicable, Celescope (v2.3.3) (https://github.com/singleron-RD/CeleScope). Reads were aligned to the *Sus scrofa* reference genome (*GCA_000003025.6*) with the corresponding Ensembl gene annotation (release 109). For each library, cell barcodes and UMIs were extracted and corrected with the chemistry-specific whitelist, and invalid barcodes were discarded. UMIs were collapsed per gene per cell, and gene-cell count matrices and position-sorted BAM files were generated using default cell-calling parameters.

### SNP calling from single-nucleus RNA sequencing data

Per-sample BAM files were subjected to SNP calling. Subsequently, two bulk-based callers (FreeBayes v1.3.8 [44] and GATK HaplotypeCaller) and four callers (Monopogen v1.6.0 [45], CellSNP-lite v1.2.3 scAllele v0.0.9.4 [46], and SCcaller v2.0.0 [47]) purpose-built for single-cell transcriptomic data were employed. For GATK HaplotypeCaller, reads were preprocessed before splitting according to the recommended best practices. CellSNP-lite was executed in pseudo-bulk mode (1b). All tools were run with their respective default or recommended parameters. To evaluate the accuracy of variant detection, SNP genotypes from matched WGS data were used as the gold standard, and benchmarking was performed using hap.py v0.3.12 (https://github.com/Illumina/hap.py).

### Pre-phasing and imputation strategies for snRNA-seq

Because snRNA-seq captures pre-mRNA and is prone to allelic dropout, it yields extremely sparse allelic depths and numerous missing genotypes, which undermines direct SNP calling from transcript data. To ensure imputation accuracy, we implemented a genotype refinement strategy and benchmarked four pipelines: (i) Beagle [48] + Beagle (both steps with Beagle 5.4), (ii) SHAPEIT2 [49] + Beagle (pre-phasing followed by imputation), (iii) SHAPEIT2 + IMPUTE5 [50], and (iv) the PGIDB online workflow [51], which also uses Beagle 5.4 for both pre-phasing and imputation. The only difference between pipeline (i) and (iv) lies in the imputation reference panel: pipeline (i) employs a panel of 953 high-quality genotypes from our prior study (Table S2) [52], whereas pipeline (iv) uses its built-in panel. We combined individual single-cell samples with the corresponding overall dataset, retaining only loci covered by single cells for phasing and interpolation. Variants were filtered according to the criteria of dosage R-squared (DR^2^) > 0.95 and minor allele frequency (MAF) ≥ 0.05. Imputation performance was assessed using concordance rate (CR) between hard calls and the truth set, the squared correlation between imputed dosages and true 0/1/2 genotypes (R^2^), as well as precision and recall.

### SNP enrichment analysis

To assess the preferential distribution of SNPs across functional genomic regions, we performed an enrichment analysis using a permutation-based approach. The analysis was designed to evaluate whether SNPs were significantly over-represented or under-represented in specific genomic compartments compared to a random distribution background. Statistical significance was assessed using a permutation test with 1,000 iterations.

### Quality control, dimension reduction, and clustering for cell type identification

Each sample was subsequently decontaminated using the celda package (v1.22.0). Cells with fewer than 500 or more than 15,000 UMIs, fewer than 200 or more than 5,000 detected genes, or with > 5% of reads mapped to mitochondrial genes were excluded from further analysis. DoubletFinder (v2.0.6) [53] was applied to identify and remove potential doublets, with the expected doublet rate set to 0.02. Quality-controlled cells were globally normalized using the Seurat (v5.2.1) [54] package.

Gene expression values were normalized with the NormalizeData function, and the top 3,000 highly variable genes were selected based on variance-stabilizing transformation. The selected genes were z-score standardized and subjected to principal component analysis (PCA). The top 30 principal components were used for clustering cells via the FindClusters function with resolution parameter set to 0.1, and the resulting clusters were visualized using uniform manifold approximation and projection (UMAP).

Given the complex sample origins, five batch correction methods were employed for data integration, including Harmony (v1.2.3) [55], scVI (v1.3.3) [56], and three approaches implemented in Seurat v5 (FastMNN, CCA, and RPCA). Batch effects were mainly introduced by differences in study origin, sequencing technology, and sequencing platform across datasets. The performance of these integration methods was quantitatively evaluated using scIB (v1.1.7) [57].

### Functional enrichment analysis

For each cell type, genes were ranked according to their coefficients of variation (CV) in expression. The top 1,000 and bottom 1,000 genes were defined as the high-variability and low-variability gene sets, respectively. BioMart (v2.60.1) [58] was used to convert Ensembl gene IDs to gene symbols, and the org.Ss.eg.db annotation package was applied to map gene symbols to Entrez IDs for downstream analyses.

Functional enrichment analyses were performed for both gene sets using the clusterProfiler (v4.12.6) [59] package. A significance threshold of adjusted *P*-value (*P*.adjust) < 0.05 was used to identify significantly enriched Gene Ontology (GO) terms.

### Pseudo-bulk eQTL analysis

Using Seurat-normalized counts, per-sample mean expression was computed for all genes, and *cis*-eQTL mapping was performed only for genes expressed (UMI > 0) in at least 1% of cells. *Cis*-eQTL mapping was performed using OmiGA (v1.0.2) [60] within a 1-megabase (Mb) window centered on the transcription start site (TSS) of each gene. Genes with a *pval_g1_acat* value below 0.05 after multiple testing correction by the permutation-free aggregated Cauchy association test (ACAT) [61] were defined as significant eGenes. For subsequent analyses, we focused exclusively on the lead SNP for each identified eGene.

Covariate selection was performed within the OmiGA framework using the parameter “--dprop-pc-covar 0.001”, which applies an elbow-based procedure to determine the number of principal components (PCs) used as covariates. Specifically, if *p*_*n*_ denotes the proportion of variance explained (PVE) by the *n*^th^ PC, OmiGA selects the first *n* components when either *p*_*n*_* − p*_*n*+1_ > *d* or *p*_*n*_ − *p*_*n*+2_ > 2*d*, where *d* = 0.001.

The replicability of our identified eQTLs was quantified using the π_1_ statistic by leveraging significant associations from the PigGTEx muscle dataset. Gene expression data for these replicated eQTLs were then downloaded from PigGTEx to validate their expression patterns.

### Single-cell eQTL analysis

*Cis*-eQTL mapping at cell-type resolution was likewise performed with OmiGA, using the same window and significance criteria. For each cell type, Seurat-normalized counts were aggregated to per-sample means. Genes were tested only if expressed (UMI > 0) in ≥ 1% of cells of that type, and sample-cell-type strata were included only when ≥ 5 cells of that type were captured. Sc-eQTL were defined as SNP-gene pairs that met the significance threshold in a given cell type. To further investigate the phenotypic impact of the candidate *cis*-eQTLs identified through our PigGTEx replicability screen, we subsequently analyzed phenome-wide association data from PigBioBank.

## Results

### Benchmarking of variant callers for snRNA-seq

**Fig. 1:**
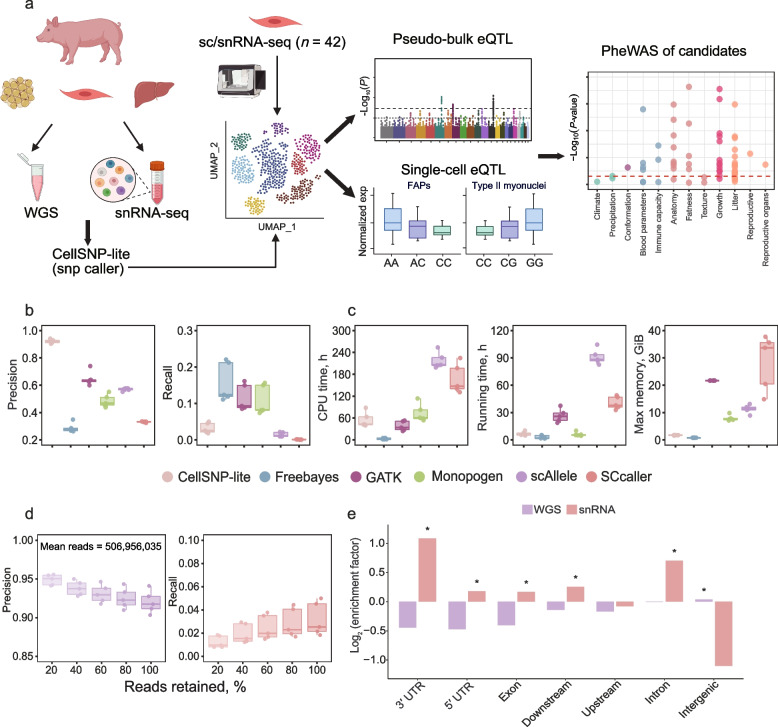
Benchmarking of SNP callers on porcine skeletal muscle snRNA-seq data. **a** Overview of the study workflow: Firstly, based on the generation of the whole genome sequencing (WGS) truth set, benchmark comparisons of six single-nucleotide variant (SNV) callers on snRNA-seq data were conducted, and the genotype data were obtained after imputation. Then, a pig skeletal muscle atlas was constructed, and *cis*-eQTL mapping (pseudo-bulk and single-cell) was performed on this basis. Finally, a PigBiobank phenotype-wide association study (PheWAS) was carried out to identify candidate regulatory variants associated with meat quality and growth traits. **b** Boxplots showing the precision and recall of six variant callers evaluated against the WGS truth set. The center line in each box represents the median, and the lower and upper hinges correspond to the first and third quartiles, respectively. **c** Boxplots displaying the computational performance of the six callers, including CPU time, wall time, and peak memory usage. **d** Boxplots showing the precision and recall of cellsnp-lite across uniformly down-sampled read depths. **e** The bar graph illustrates the enrichment of SNPs in functional genomic regions from WGS and snRNA-seq data

To ensure accurate downstream analyses of RNA-derived genotyping and eQTL mapping, we first established a high-confidence SNP truth set from WGS data. We selected five samples matched to the snRNA-seq cohort and processed their WGS data according to the GATK4 Best Practices for germline short-variant discovery (Fig. 1a), achieving a mean coverage breadth of 98% and a mean depth of 23 × (Table S3). After hard filtering, the truth set comprised 17,264,159 high-quality variants. Variant counts were highest on chromosomes 1 and 13, whereas per-kilobase variant density was lowest on chromosome 1, consistent with its larger physical size (Fig. S1a and Table S4). As expected, transitions outnumbered transversions (Ti/Tv > 1; Fig. S1b and Table S5), which is a widely observed feature of germline SNP spectra and a standard WGS quality-control indicator [62, 63]. This variant call set was then used as the reference for benchmarking the RNA-based SNP callers in subsequent analyses.

We benchmarked six SNP callers (CellSNP-lite, FreeBayes, GATK, Monopogen, scAllele, and SCcaller) on snRNA-seq data, using the matched WGS call set as the reference. Using precision and recall as evaluation metrics, we observed markedly different performance profiles across the six tools (Fig. 1b and Table S6). CellSNP-lite delivered the highest precision (consistently > 0.9) but lower recall, reflecting a conservative model that favors specificity under UMI-aware conditions with low read depth. In contrast, FreeBayes achieved relatively high recall among the bulk-oriented callers but at the cost of lower precision, indicating more false positives on sparse nuclear RNA data. GATK showed intermediate performance, whereas SCcaller and scAllele performed poorly for both precision and recall. Except for Monopogen, the evaluation results for these callers were broadly consistent with those reported in other studies [45, 64]. The reduced precision of Monopogen likely stems from its default assumptions and parameter settings, which are optimized for human data (e.g., reference resources and LD scale) and are not fully portable to the porcine genomic context.

To investigate which SNP calling tool is more efficient, we further evaluated the computational resource usage of these six software tools (Fig. 1c and Table S7). We found that CellSNP-lite and FreeBayes incurred shorter CPU and wall times, and maintained the lowest peak memory usage. Monopogen showed intermediate resource consumption, scAllele required the longest CPU and wall times, and SCcaller and GATK exhibited the highest peak memory usage. Taken together, these results highlight CellSNP-lite as a favorable operating point, combining high precision with low computational cost for SNP calling. Accordingly, we adopted CellSNP-lite for subsequent SNP genotyping throughout the atlas.

To assess the effect of sequencing depth on SNP calling, we uniformly subsampled each library to 20%, 40%, 60%, and 80% of the original reads (using a fixed random seed) and recomputed precision and recall. Recall increased with greater read retention, whereas precision decreased modestly (Fig. 1d and Table S8). This pattern reflects the typical trade-off between sensitivity and specificity, because additional depth recovers more true positives but also introduces a larger number of low-support candidates, similar to observations from independent variant-calling benchmarks [65].

To characterize the genomic distribution of SNPs derived from snRNA-seq, we first annotated variant positions by genic context (intergenic, intronic, exonic, 5'/3' UTR, upstream, downstream) and compared these patterns with WGS-derived variants. Consistent with prior reports [45, 66], most WGS variants localized to introns (57.6%) and intergenic regions (27.7%), whereas variants called from snRNA-seq were predominantly intronic (75.9%) (Fig. S2 and Table S9). Relative to the genomic background, snRNA-seq calls showed strong enrichment in introns and 3' UTRs region, accompanied by a reciprocal depletion in intergenic regions, whereas WGS displayed the opposite tendency (Fig. 1e and Table S10). This pattern is expected in single-nucleus transcriptomic assays, as these protocols capture abundant pre-mRNA, thereby yielding many intronic alignments and enriched 3' UTR reads. Accordingly, the inclusion of an intronic signal is a standard practice for snRNA-seq quantification [67].

### Evaluating pre-phasing and imputation strategies for variants from snRNA-seq

To recover missing genotypes caused by sparse allelic depth in sn/scRNA-seq data, we leveraged linkage disequilibrium (LD) patterns by performing pre-phasing and imputation. We benchmarked four pipelines and evaluated imputation performance against a matched WGS truth set.

**Fig. 2:**
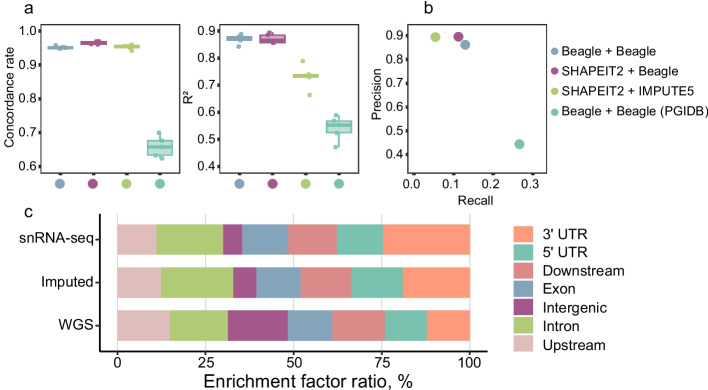
Genotype imputation evaluation for sc/snRNA-seq data. **a** Genotype refinement for snRNA-seq data. Boxplots summarize the CR and R^2^ for Beagle + Beagle, SHAPEIT2 + Beagle, SHAPEIT2 + IMPUTE5, and Beagle + Beagle (PGIDB). **b** Scatter plot showing the precision versus recall of the four imputation methods against the WGS truth set. **c** The bar chart showing the preference distribution of the three SNP set categories in the functional genomic regions

Across our evaluations, the three offline workflows were stable, with CR values greater than 0.90 and R^2^ in the 0.80–0.90 range. SHAPEIT2 + Beagle achieved the highest CR, whereas SHAPEIT2 + IMPUTE5 achieved comparable CR but slightly lower R^2^ under our reference panel and parameterization. In contrast, the PGIDB web pipeline yielded higher recall but lower precision, R^2^, and CR compared with the offline workflows. This difference likely arises from the use of a fixed public reference panel and default parameter settings in the online pipeline, which are designed to maximize variant recovery across diverse populations but may be less well matched to the genetic background and genome assembly of our cohort. Moreover, the absence of additional post-imputation filtering, such as DR^2^ or MAF thresholds, further favors recall at the expense of precision (Fig. 2a, b and Table S11). These trends are consistent with prior evaluations, which have shown that pre-phasing combined with LD-aware imputation (e.g., SHAPEIT2 + Beagle) can achieve high accuracy even at low depth [50, 68]. Given its superior CR and precision, the SHAPEIT2 + Beagle combination was selected for all subsequent analyses.

We compared the genomic distribution of variants before imputation (direct snRNA-seq calls) and after imputation. Although both sets displayed the characteristic snRNA-seq pattern with enrichment in 3’ UTRs and introns and depletion in intergenic regions (Fig. 2c and Table S10), the imputed set shifted toward the WGS background, with a lower fraction of 3’ UTR calls, reduced exonic representation, and a modest increase in intergenic sites. This movement toward the genomic baseline is consistent with LD-aware imputation, which introduces variants beyond expressed loci and thus broadens genomic coverage relative to 3’-end, pre-mRNA-rich calls.

### Characterization of skeletal muscle cell populations

**Fig. 3:**
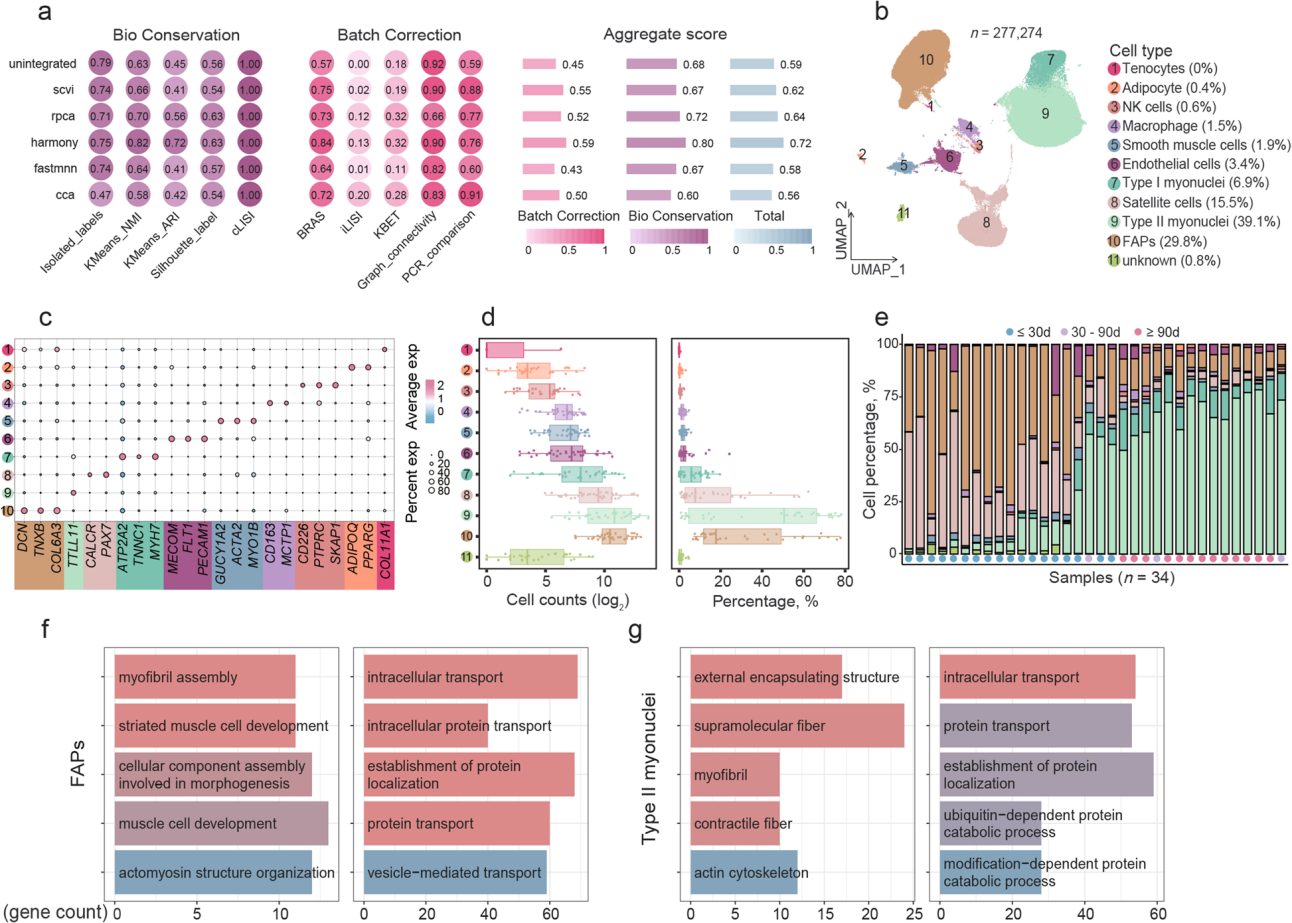
Characterization of skeletal muscle cellular heterogeneity. **a** Benchmarking of five integration methods for batch effect correction across different studies. **b** UMAP visualization of 277,274 nuclei from all individuals, colored by ten distinct cell types annotated to their respective lineages. **c** Dot plot of canonical marker genes for each cell lineage. Color intensity represents the average expression, and dot size indicates the percentage of cells expressing the gene. **d** Boxplots showing the absolute cell numbers (left) and proportional abundances (right) for each cell type across samples. **e** Stacked bar plot depicting the proportional abundance of each cell type across samples, with corresponding age information. **f** GO enrichment analysis for the top 1,000 most variable (high CV) and least variable (low CV) genes in FAPs. **g** The same analysis as in (**f**), performed in type II myonuclei

To elucidate the cell-type-specific gene regulation underlying skeletal muscle development and meat production traits, we first constructed a single-cell atlas of porcine skeletal muscle. To this end, we integrated 36 publicly available sc/snRNA-seq samples with our six newly generated datasets, thereby forming a combined cohort of 42 samples spanning 12 breeds for downstream analyses. After stringent quality control, 34 eligible samples were retained, which spanned embryonic to adult stages and yielded 277,274 high-quality cells/nuclei. Since data from different studies often exhibit batch effects, we used the scIB benchmarking framework to evaluate five commonly used integration methods, including Seurat CCA, RPCA, fastMNN, scVI, and Harmony, with the goal of mitigating cross-study batch effects. Our evaluation revealed that Harmony achieved the highest aggregate score on our scIB benchmark, with a total score of 0.72, a biological conservation score of 0.80, and a batch correction score of 0.59 (Fig. 3a and Table S12). This result indicates that Harmony provides a favorable balance between batch effect removal and biological conservation, so it was selected for downstream analyses. The UMAP based on the Harmony embedding showed good mixing of cells across studies and assay types (Fig. 3b and Fig. S3a, b), confirming successful integration while preserving the biological signal.

Using well-characterized cell-type-specific marker genes, we identified ten major cell types in the integrated dataset (Fig. 3c), including fibro/adipogenic progenitors (FAPs), type I myonuclei, type II myonuclei, satellite cells, endothelial cells, smooth muscle cells, macrophages, adipocytes, tenocytes, and natural killer (NK) cells. We systematically quantified cell abundance across all samples to examine how cellular composition varied with developmental stage. The atlas was characterized by a high abundance of type II myonuclei (39.1%) and FAPs (29.8%), which together formed the majority population. This skewed distribution was apparent in both the global and per-sample assessments (Fig. 3b, d, and Table S13). Furthermore, we identified a clear age-dependent shift in cellular composition, despite a background of inter-individual variability (Fig. 3e and Table S12). Muscles from samples ≤ 30 d were enriched for FAPs and satellite cells, indicative of progenitor-rich, remodeling tissue, whereas samples ≥ 90 d were dominated by type II myonuclei, consistent with postnatal maturation and fiber hypertrophy. This progressive transition from a progenitor-supported to a myonuclear-dominated state is consistent with established muscle biology.

To probe the cellular functions responsible for inter-sample variability, we computed the gene-wise coefficient of variation (CV) within each cell type using Seurat-normalized counts, and then performed GO Biological Process enrichment on the top and bottom 1,000 genes ranked by CV. Because FAPs and type II myonuclei were consistently present across all samples and comprised sufficient numbers of cells, they enabled the derivation of more stable and reproducible cell-type-specific transcriptional profiles. In addition, both cell types are closely linked to muscle development, metabolism, and fat deposition. We therefore focused on these two cell types in the following analyses. In FAPs, high-variance genes were enriched for terms related to myofibril assembly and muscle development, whereas low-variance genes mapped to intracellular transport pathways (Fig. 3f). In type II myonuclei, high-variance genes highlighted terms associated with contractile fibers and myofibrils, while low-variance genes again pointing to core transport and proteostasis functions (Fig. 3g). These patterns suggest dynamic regulation of myogenic and structural programs superimposed on a stable housekeeping backbone within each lineage, consistent with the roles of FAPs in orchestrating myogenesis [69] and of type II myonuclei in contractile specialization [70].

### Pseudo-bulk eQTLs in skeletal muscle and cross-dataset replication

**Fig. 4:**
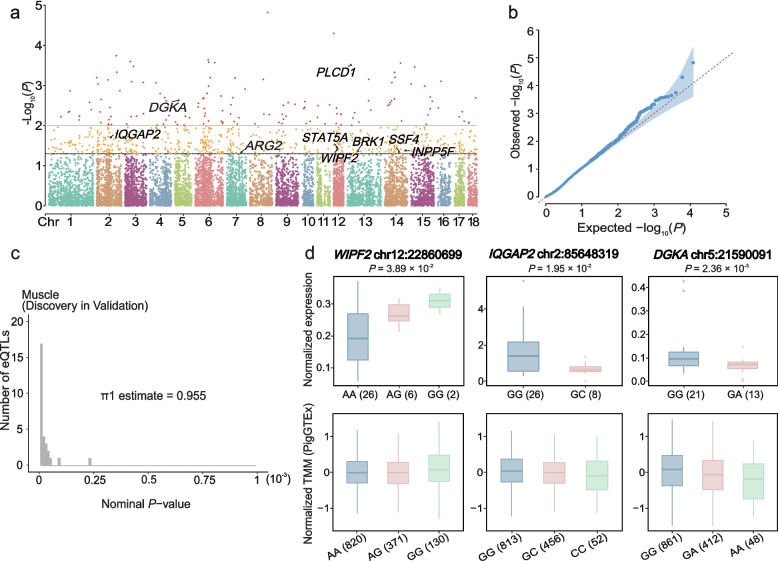
Pseudo-bulk *cis*-eQTL mapping in porcine skeletal muscle. **a** Manhattan plot of *cis*-eQTL associations. Each point represents a variant. The black and grey horizontal lines denote significance thresholds at *P* = 0.05 and *P* = 0.01, respectively. Points are colored red (*P* < 0.01) or yellow (0.01 ≤ *P* < 0.05) based on the *pval_g1_acat*. Key genes related to muscle biology (e.g., *PLCG1*, *DGKA*, and *IQGAP2*) are annotated. **b** Q-Q plot of observed versus expected −log_10_(*P*-values) for *cis*-eQTL associations. The red dashed line represents the null hypothesis. **c** Replication rate (π₁) of significant *cis*-eQTLs in an external PigGTEx validation dataset. **d** Comparison of genotype-dependent expression patterns between the internal dataset and external PigGTEx validation data. Top: Expression quantitative trait loci (eQTL) effects for *WIF1* (chr12:22860699), *IQGAP2* (chr8:28548319), and *DGKA* (chr5:21550091) in the discovery cohort. Bottom: Corresponding expression patterns from the PigGTEx database

Since tissue-level eQTL mapping is well established, we first validated our workflow at the pseudo-bulk tissue level. Sample-wise sc/snRNA-seq counts were aggregated to gene-level expression, and *cis* associations were tested within ±1 Mb of each TSS, with one lead SNP per gene selected for display. In total, we detected 625 significant *cis*-eQTLs after correction for multiple testing (Fig. 4a and Table S14). The Manhattan plot revealed widespread *cis*-regulatory signals across the genome, with representative loci such as *IQGAP2*, *DGKA*, and *PLCD1* surpassing the significance line. In a complementary view, the Q-Q plot tracked the null expectation over most of the distribution with a pronounced tail exceeding the 95% confidence band (Fig. 4b), which indicates true polygenic enrichment rather than systematic inflation.

Furthermore, we performed direction-aware replication against PigGTEx skeletal-muscle *cis*-eQTLs [6]. Using significant SNP-gene pairs from PigGTEx as the discovery set, we extracted their nominal *P*-values from our data and estimated the proportion of non-null effects with Storey’s π₁ [71]. The replication histogram showed the expected pile-up near zero, and yielded a π₁ estimate of 0.955 (Fig. 4c), which supports the high reproducibility between our results and the PigGTEx reference. To further illustrate this concordance, we highlighted several representative SNP-gene pairs related to skeletal muscle development and production traits, such as *WIPF2*, *IQGAP2*, and *DGKA*. For each case, the genotype-stratified expression in our cohort recapitulated the specific direction and allele dosage trend observed in PigGTEx (Fig. 4d). Taken together, these consistent findings demonstrate the overall accuracy and robustness of the framework that we established, even though the sample size was limited.

### Comprehensive identification and characterization of single-cell eQTLs

**Fig. 5:**
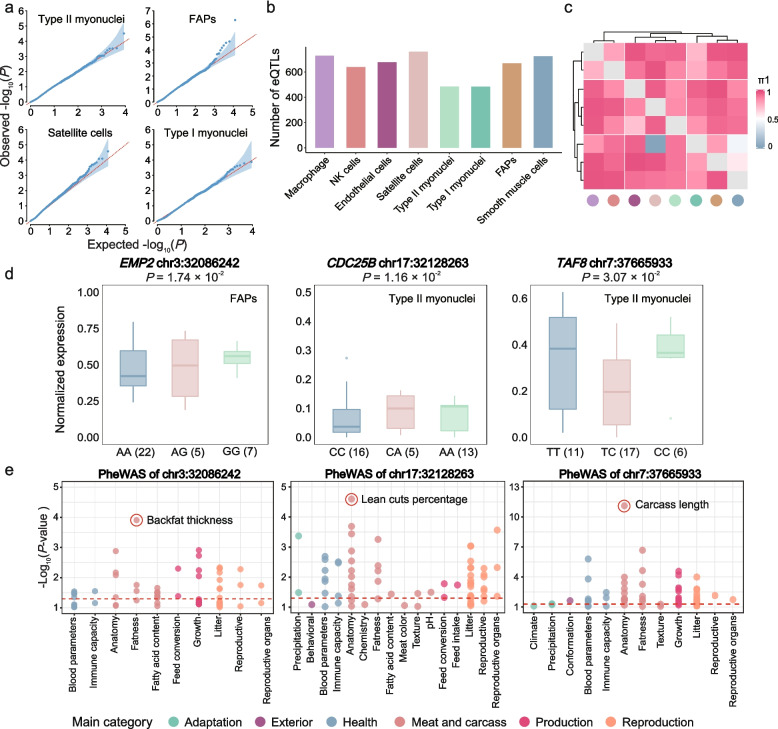
Single-cell *cis*-eQTL analysis. **a** Q-Q plots of *cis*-eQTL *P*-values for four representative cell types. **b** The bar chart showing the number of significant eQTLs in each cell type. **c** Hierarchically clustered heatmap of the directional π₁ matrix (rows = discovery, columns = replication); independent row/column clustering using Pearson correlation distance (1 − *r*) and average linkage (pairwise-complete observations). The color of the dot below corresponds to (**b**). **d** Single-cell *cis*-eQTL examples. Box plots show genotype-expression associations for *EMP2* in FAPs (chr3:32086242), *CDC25B* (chr17:32128263) and *TAF8* (chr7:37665933) in Type II myonuclei. **e** Phenome-wide association study (PheWAS) of candidate *cis*-eQTLs. The plots show the association significance (−log_10_(*P*-value)) for the lead variants of *EMP2*, *CDC25B*, and *TAF8* across various phenotypic categories. The red dashed line indicates the significance threshold (*P* = 0.05), and significantly associated traits are highlighted with red circles

To uncover cell-type-specific regulatory variants, we performed direct sc-eQTL mapping across eight cell types with relatively large sample sizes. Across cell types, Q-Q plots showed a systematic upward deviation of observed from expected −log_10_(*P*) values and exhibited tail inflation beyond the 95% confidence band (Fig. 5a and Fig. S4). After multiple-testing correction (pval_g1_acat < 0.05), we identified 5,020 sc-eQTLs in total, with a mean of 628 per cell type (range 475–696) (Fig. 5b and Table S15).

To assess the extent of *cis*-eQTL sharing across cell types, we performed hierarchical clustering based on the π₁ statistic (Fig. 5c and Table S16). This analysis delineated three distinct co-sharing modules: (i) an immune module clustering NK cells with macrophages, reflecting their functional relatedness through a high degree of shared genetic regulation, (ii) a progenitor/fast-fiber module centered on satellite cells and type II myonuclei, and (iii) a slow-fiber/stromal module comprising type I myonuclei, fibro-adipogenic progenitors (FAPs), and smooth muscle. Overall, *cis*-eQTL sharing was substantially stronger within these modules than between them.

Building on prior evidence from PigGTEx and single-cell studies [22, 72–74], which suggest that sc-eQTLs are associated with complex traits, we further investigated sc-eQTLs that may have a potential impact on meat production. We prioritized sc-eQTLs that displayed clear genotype-expression gradients (Fig. 5d) and evaluated their phenome-wide associations in the PigBiobank datasets (Fig. 5e). Three representative loci showed significant phenome-wide associations within major categories of meat quality and carcass composition, providing phenotypic support that prioritizes these sc-eQTLs as candidates for further functional investigation. In FAPs, the chr3:32086242 locus in *EMP2* displayed an allele dosage dependent trend in expression and showed the highest PheWAS association signal for backfat thickness within the “Fatness” subcategory. In type II myonuclei, the *CDC25B* eQTL (chr17:32128263) showed clear genotype stratification (CA/AA > CC) and was significantly associated with lean cuts percentage in the “Anatomy” subcategory. Similarly, the chr7:37665933 locus in *TAF8* also presented an ordered expression gradient and yielded the top association signal for carcass length under the “Anatomy” subcategory. Taken together, these PheWAS results provide functional support and trait-based prioritization for selected sc-eQTLs, highlighting candidate regulatory loci with potential relevance to economically important traits and serving as a valuable resource for informing future genomic selection and precision genome editing in pigs.

## Discussion

This study integrates sc/snRNA-seq data to construct a comprehensive atlas of porcine skeletal muscle, enabling the characterization of a single-cell eQTL landscape related to meat quality and carcass composition. Beyond establishing a benchmarking workflow for variant detection and genotype imputation tailored to single-cell transcriptomic data in pigs, we highlight how non-coding genetic variation influences economically important traits via cell-type-specific transcriptional regulation. Our work thus provides both a methodological foundation and a set of actionable candidate regulatory variants for molecular breeding.

Single-cell and single-nucleus transcriptomes exhibit extremely sparse coverage, a strong 3’ bias, and abundant pre-mRNA/intronic reads, yielding an evidence model that is fundamentally different from that of bulk RNA-seq or WGS [75]. Consequently, the direct application of bulk-oriented SNP callers to sc/snRNA-seq data often disrupts the balance between sensitivity and specificity or increases the false-positive rate. To address these limitations, we established a pig-specific workflow aligned with the data-generation mechanism. Given the trade-off among sensitivity, specificity, and computational resources, we used CellSNP-lite as the core per-cell genotyper because of its favorable precision and scalability. Its UMI-aware, locus-by-cell counting and filtering scheme, optimized for droplet-based single-cell libraries, maintains high precision under low coverage and biased read distributions (precision consistently ≥ 0.9 in our benchmarks). Although recall is conservative, the resulting allele-count matrices are more reliable for eQTL. Notably, while Monopogen demonstrates strong performance on human datasets by leveraging extensive haplotype and LD resources, its transferability to porcine data may be limited. Differences in the LD architecture between pig and human, coupled with comparatively less comprehensive porcine reference panels compared to human resources like the 1000 Genomes Project and gnomAD, likely contribute to the observed reductions in accuracy and consistency [76, 77]. Overall, the implementation of single-cell genotyping and eQTL mapping in livestock species, particularly pigs, benefits from workflows that are both species-aware and single-cell-aware. Such workflows should begin with a high-precision core set of variants and incorporate species-specific LD modeling and reference-panel optimization to balance accuracy, scalability, and biological interpretability.

Across developmental stages, the integrated atlas recapitulates a compositional shift consistent with classical myogenesis, characterized by an enrichment of FAPs and satellite cells at early stages (≤ 30 d) and an eventual dominance of type II myonuclei at ≥ 90 d. This age-linked structural and cytological transition is consistent with a model in which FAPs support satellite cell activation and differentiation through paracrine signaling, thereby coordinating developmental and regenerative myogenesis [78]. In pigs, postnatal muscle growth primarily reflects fiber hypertrophy, and metabolic and contractile programs progressively shift toward adult-type fast (type II) fibers, consistent with the observed increase in type II myonuclei [70, 79]. Functionally, genes with high expression variability were linked to specialized pathways that are subject to regulation, whereas low-variance genes were associated with fundamental housekeeping processes. This pattern, in which modules that are subject to regulation exhibit greater cell-to-cell dispersion whereas housekeeping programs remain stable, is well supported by previous studies [80–82].

At the eQTL level, many single-cell studies still rely on deconvolving cell-type fractions from bulk tissues and testing genotype-by-fraction interactions, largely due to the high cost of population-scale sc/snRNA-seq [83–86]. Although economical and informative, this strategy is sensitive to reference-atlas matching, inter-individual variability, and model specification, and it has limited power to detect eQTLs in rare or transitional cell states [87]. These limitations underscore the need for direct single-cell eQTL mapping when feasible. Although statistical power in sc-eQTL mapping is strongly influenced by sample size [88], several exploratory studies have demonstrated the feasibility of cell-type-resolved regulatory analyses using moderately sized cohorts. Notably, a number of published sc-eQTL and related single-cell QTL studies across diverse tissues and regulatory contexts have been conducted with sample sizes ranging from approximately 39 to 89 individuals [13, 89–92]. In this context, our study adopted a pragmatic design to address the practical constraint of limited sample availability. By integrating a multi-breed single-cell atlas with stringent QC, high-precision genotyping, and pig-specific imputation, we established a robust framework for identifying sc-eQTLs in pigs.

Within this framework, we identified approximately 628 significant *cis*-eQTLs for each cell type on average, most of which exhibit clear cell-type anchoring, and we nominate several candidates that plausibly link non-coding variants to economically relevant phenotypes through gene-expression modulation. In FAPs, an *EMP2* eQTL shows a dosage-graded effect on *EMP2* expression. *EMP2* encodes a four-pass epithelial membrane protein that regulates caveolae homeostasis and integrin recycling [93, 94]. Perturbations along the caveola-integrin axis modulate mechanosensation as well as cell adhesion and migration, both of which are processes relevant to intramuscular adipogenesis and matrix remodeling [95, 96]. Altered *EMP2* levels in progenitors thus provide a tractable mechanistic path from non-coding variation to progenitor behavior and fat deposition, consistent with the observed association with backfat thickness in PheWAS. In type II myonuclei, allelic variation at the *CDC25B* locus is associated with the percentage of lean cuts, whereas variation at the *TAF8* locus is associated with carcass length, with both loci acting through analogous cell-type-specific regulatory mechanisms [97, 98]. Collectively, these candidates illustrate how cell-type-specific regulatory variation can provide biologically interpretable links between non-coding variants and complex traits, while PheWAS results serve to support functional relevance and prioritize loci for further validation. From an applied standpoint, such sc-eQTL constitute prioritized markers for genomic selection and testable targets for functional validation, with clear potential to influence meat quality, carcass composition, and production efficiency.

Despite these significant findings, several limitations should be noted. Firstly, our variant discovery relied solely on RNA-derived genotypes, which tend to under-represent intergenic and low-expression regulatory regions relative to WGS or dedicated SNP arrays. Although stringent imputation quality filtering was applied to minimize the impact of inaccurate genotype calls, this limitation remains inherent to RNA-based variant discovery. Secondly, the high cost of sc/snRNA-seq constrained our cohort size, thereby limiting the statistical power to detect eQTLs with moderate or weak effects. In addition, although the effective sample sizes across the analyzed cell types were highly similar, not all cell types were detected in every sample, and some cell types contained fewer cells per sample. This may introduce residual differences in statistical power for sc-eQTL detection. Future studies with expanded sample sizes, improved pig-specific genomic resources (e.g. haplotype panels), and integration of multi-omics data will be crucial for uncovering the full spectrum of regulatory variation and clarifying its functional impact for breeding applications, including enabling well-powered colocalization analyses to more directly link regulatory variants with complex traits.

## Conclusion

This study demonstrates that single-cell eQTL mapping in pigs can uncover the cellular basis of complex economic traits, even with limited sample sizes. By constructing a skeletal muscle atlas, we identified key regulatory loci and linked them to phenotypes, highlighting three specific candidates associated with meat quality and growth traits. Our findings provide a scalable framework for single-cell eQTL mapping in livestock and contribute to elucidating the role of non-coding variation in shaping economically important traits, with direct implications for genomic selection and precision breeding.

## Supplementary Information

Additional file 1: Fig. S1. Quality assessment of the WGS-derived SNP truth set. a) The histogram showing the number and density of variations on different chromosomes. b) The transition-transversionmatrix heatmap. Fig. S2. The pie chart showing the distribution of SNPs in the functional genomic regions in the WGS and snRNA-seq data. Fig. S3. Assessment of batch effects before and after data integration. a–c) UMAP plots illustrating cellular distributions before batch effect correction. d–f) UMAP plots showing the corresponding embeddings after batch correction. Fig. S4. Q-Q plots of the remaining four cell types.

Additional file 2: Table S1. The public data used in this study. Table S2. Sequencing quality and read alignment statistics for the 953-sample Pig Genomics SNP Reference Panel. Table S3. The SAM tools coverage reports of each sample. Table S4. Variants rate details of WGS. Table S5. Base changes of WGS. Table S6. Benchmarking summary of variant callers on snRNA-seq. Table S7. Comparative analysis of computational resource usage across variant callers. Table S8. The precision and recall rate of CellSNP-lite when performing uniform downsampling of read depth. Table S9. the distribution of SNPs in the functional genomic regions in the WGS and snRNA-seq data. Table S10. The enrichment of the three SNP collection categories in the functional genomic regions. Table S11. Performance evaluation of imputation methods for genotype inference in sc/snRNA-seq data. Table S12. Benchmarking of five integration methods for batch effect correction across different studies. Table S13. The number of cells of each type in different samples. Table S14. Output summary statistics of the analysis of pseudo-bulk *cis*-eQTL. Table S15. Output summary statistics of the analysis of single-cell *cis*-eQTL. Table S16. Statistical analysis of the directional π₁ values among cell types.

## Data Availability

The whole genome resequencing data and newly generated snRNA-seq data that were analyzed during the current study are available from the corresponding author on reasonable request. The public sc/snRNA-seq data that were analyzed during the current study are available in the NCBI and CNCB repository. Please see Table S1 and Refs. [29–37] for details and links to the data.

## Abbreviations

ACAT: The permutation-free aggregated Cauchy association test
CR: Concordance rate
CV: Coefficient of variation
DR^2^: Dosage R-squared
eQTL: Expression quantitative trait locus
FAPs: Fibro/adipogenic progenitors
GO: Gene Ontology
GTEx: Genotype-Tissue Expression
GWAS: Genome-wide association studies
LD: Linkage disequilibrium
MAF: Minor allele frequency
NK: Natural killer
PCR: Polymerase chain reaction
PheWAS: Phenome-wide association studies
QC: Quality control
R^2^: Squared correlation between imputed dosages and 0/1/2 genotypes
scRNA-seq: Single-cell RNA sequencing
sc-eQTL: Single-cell eQTL
SNP: Single-nucleotide polymorphisms
snRNA-seq: Single-nucleus RNA sequencing
UMI: Unique molecular identifier
WGS: Whole-genome sequencing
