# Supplementary material for: A scalable framework for single-cell eQTL mapping uncovers genetic regulators of meat production traits in pigs
Source: J Anim Sci Biotechnol. 2026 Jun 28;17:132. doi: 10.1186/s40104-026-01452-5 (PMC13310442; doi:10.1186/s40104-026-01452-5)
Supplement: Supplementary file 1 — Additional file 1: Fig. S1. Quality assessment of the WGS-derived SNP truth set. a) The histogram showing the number and density of variations on different chromosomes. b) The transition-transversionmatrix heatmap. Fig. S2. The pie chart showing the distribution of SNPs in the functional genomic regions in the WGS and snRNA-seq data. Fig. S3. Assessment of batch effects before and after data integration. a–c) UMAP plots illustrating cellular distributions before batch effect correction. d–f) UMAP plots showing the corresponding embeddings after batch correction. Fig. S4. Q-Q plots of the remaining four cell types. [file 40104_2026_1452_MOESM1_ESM.docx]

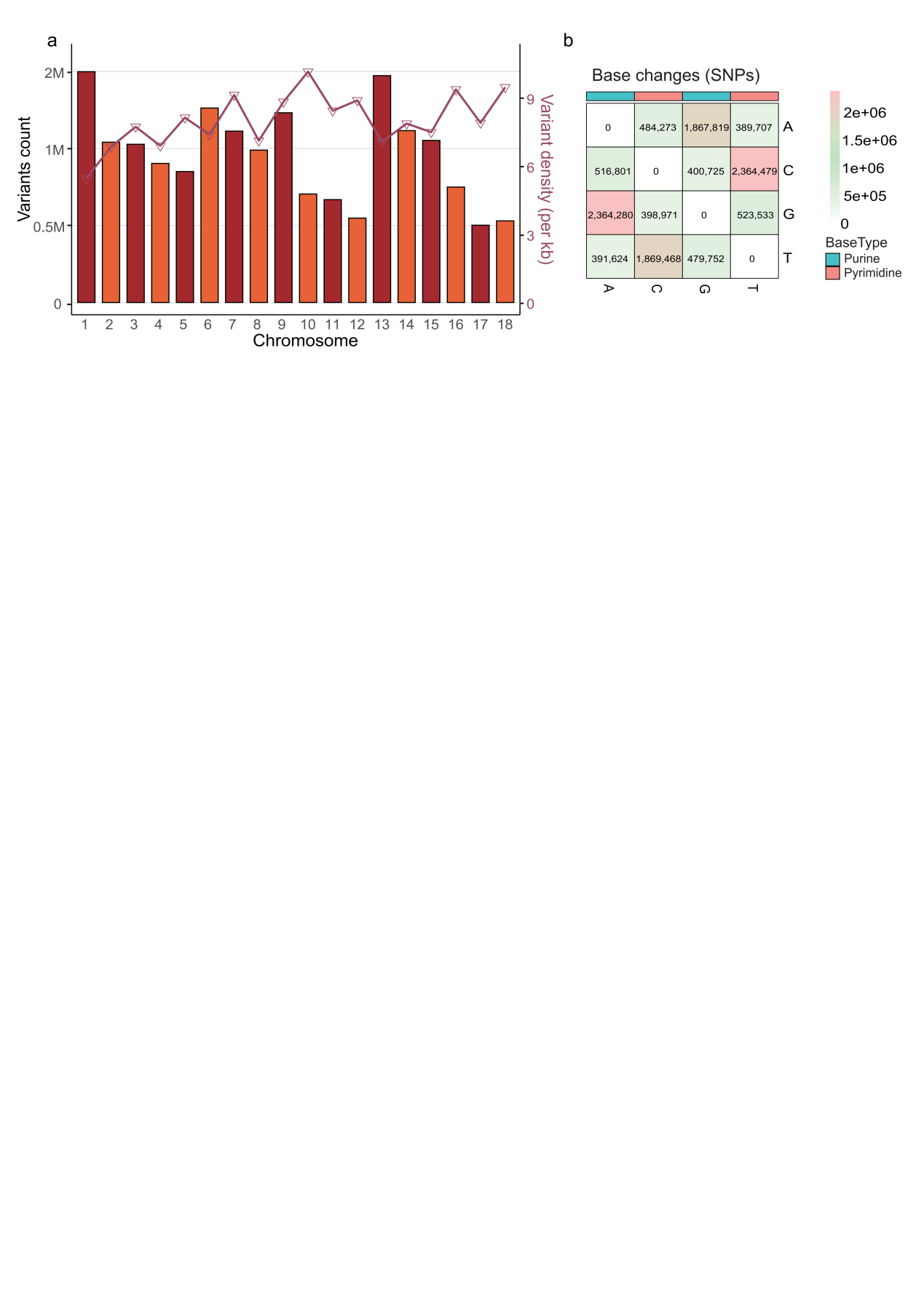


Fig. S1 Quality assessment of the WGS-derived SNP truth set. a) The histogram showing the number and density of variations on different chromosomes. b) The transition-transversion (Ti/Tv) matrix heatmap.


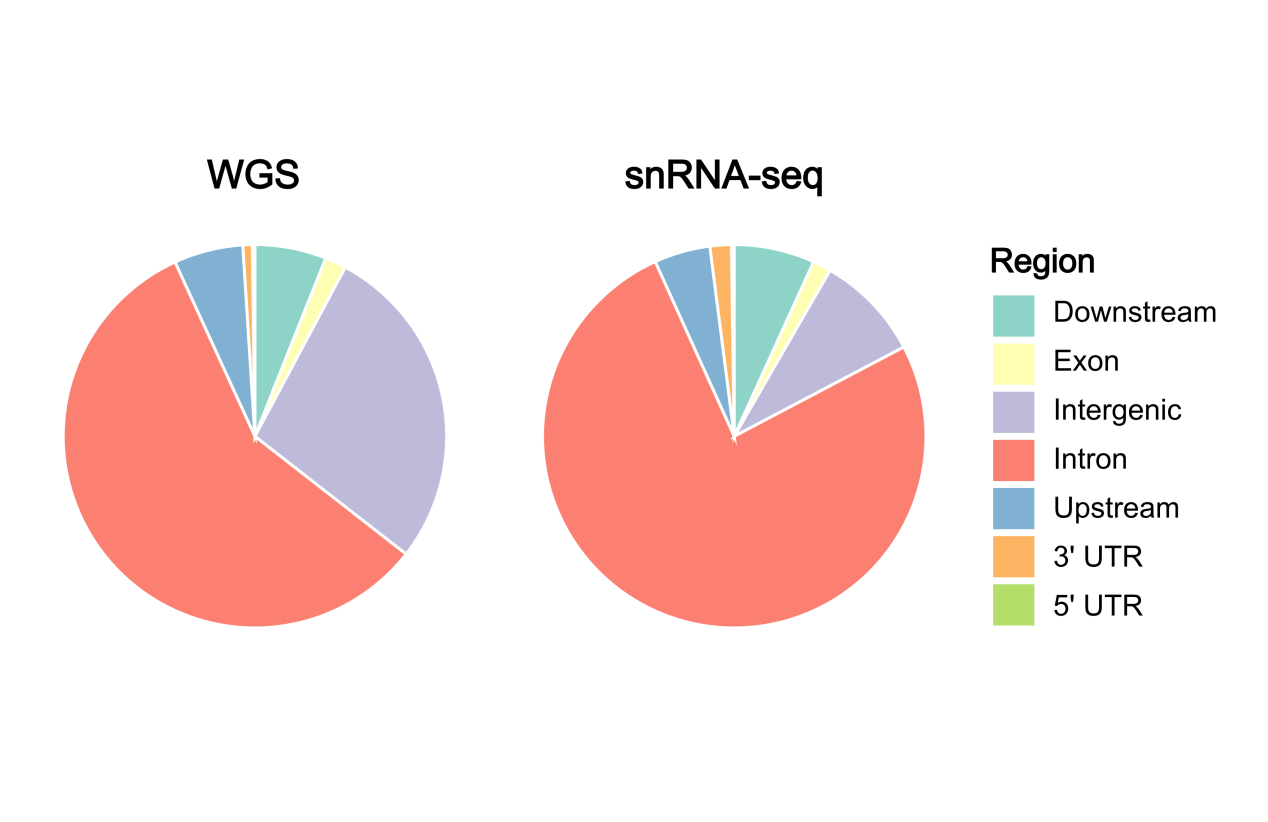
Fig. S2 The pie chart showing the distribution of SNPs in the functional genomic regions in the WGS and snRNA-seq data.


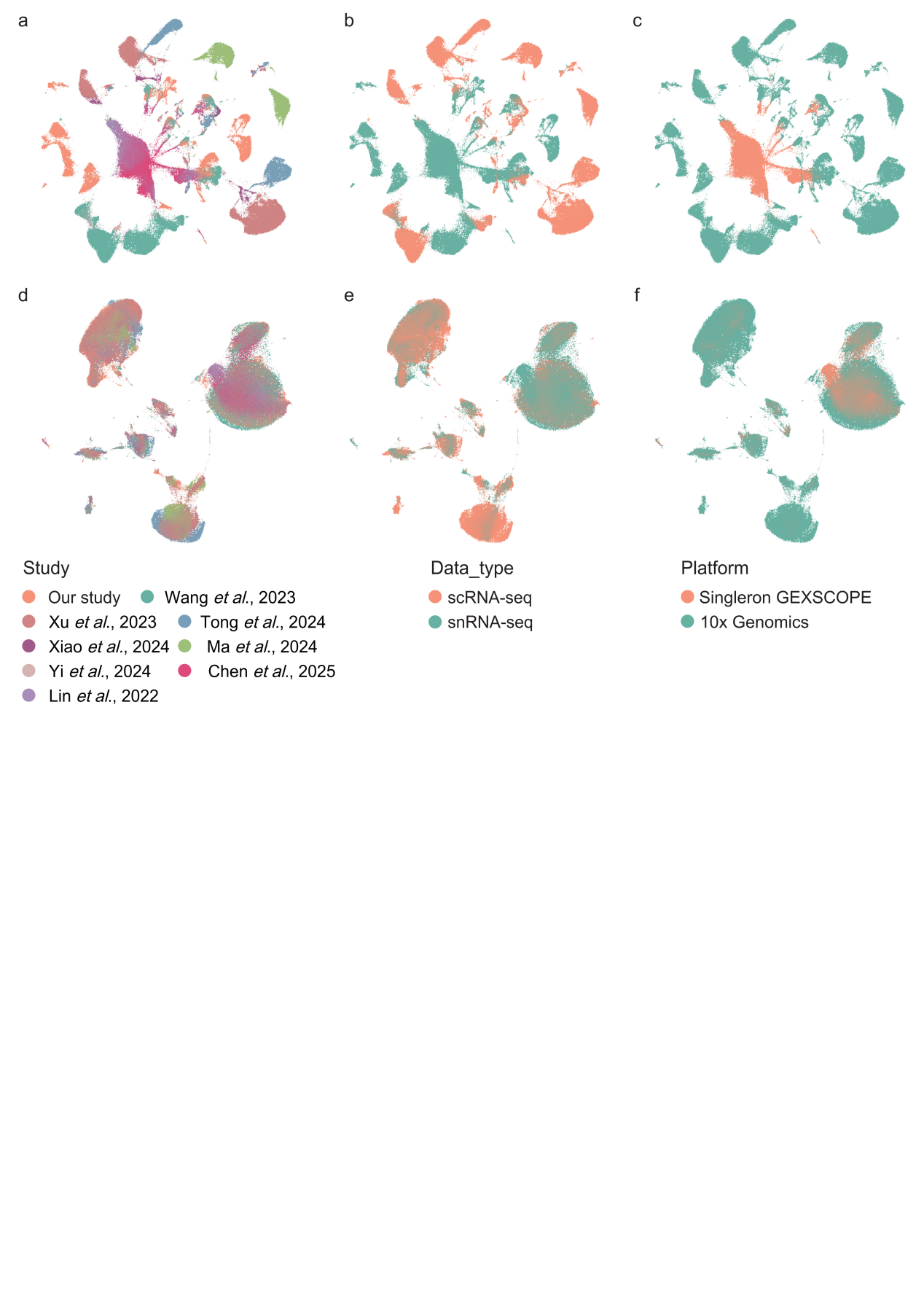


Fig. S3 Assessment of batch effects before and after data integration. a-c) UMAP plots illustrating cellular distributions before batch effect correction. d-f) UMAP plots showing the corresponding embeddings after batch correction.

Fig. S4 Q-Q plots of the remaining four cell types.
